# Fluid removal improves muscle performance and weakness in critically ill patients: a pilot study

**DOI:** 10.1186/s40635-025-00830-z

**Published:** 2025-11-24

**Authors:** Nicolás Arancibia, René López

**Affiliations:** 1 Servicio de Medicina Física y Rehabilitación, Clínica Alemana de Santiago, Santiago, Chile; 2 Departamento de Paciente Crítico, Clínica Alemana de Santiago, Santiago, Chile; 3 Grupo Intensivo, Instituto de Ciencias e Innovación en Medicina ICIM, Facultad de Medicina, Clínica Alemana—Universidad del Desarrollo, Santiago, Chile

**Keywords:** Ultrafiltration, Muscle ultrasound, ICU-acquired weakness, Renal replacement therapy, Muscle strength, Fluid overload

## Abstract

**Background:**

Fluid overload in critically ill patients has been associated with muscle edema, decreased tissue quality, and the development of intensive care unit-acquired weakness (ICU-AW). Continuous renal replacement therapy (CRRT) with ultrafiltration (UF) contributes to removing excess extracellular fluid. This study aimed to evaluate whether UF is associated with changes in muscle ultrasound parameters and strength in critically ill patients.

**Methods:**

Critically ill patients with resolved hypoperfusion undergoing CRRT with fluid removal via UF were prospectively enrolled and compared with a control group without UF. Muscle ultrasound assessments included rectus femoris and vastus intermedius thickness, echogenicity, and subcutaneous tissue. Global muscle strength was assessed using the Medical Research Council Sum Score (MRC-SS). Assessments were performed at CRRT initiation (T1) and again 36 h later (T2).

**Results:**

Twenty-eight patients were enrolled, 18 in the UF group and 10 patients in the control group. All ultrasonographic variables measured were different between the UF and control groups. In the UF group, median rectus femoris thickness decreased from 1.74 to 1.57 cm (*p* = 0.03), vastus intermedius from 1.14 to 0.95 cm (*p* < 0.01), echogenicity from 91.7 to 78.3 grayscale units (*p* < 0.01), and subcutaneous tissue thickness from 1.98 to 1.79 cm (*p* < 0.01). MRC-SS increased from 45.0 to 49.0 points (*p* = 0.05). A positive correlation was found between UF volume (mL/kg) and MRC-SS at T2 (*ρ* = 0.71, *p* < 0.01), and a negative correlation between UF volume and change in muscle echogenicity (*ρ* = − 0.49, *p* = 0.039). ROC curve analysis identified that a UF volume ≥ 82 mL/kg was associated with MRC-SS > 48 points obtaining an AUC of 0.982 (95% CI: 0.928–1.000), sensitivity 92.9%, and specificity 100%.

**Conclusion:**

Ultrafiltration was associated with changes in muscle echogenicity and subcutaneous tissue as well as an increase in MRC scoring at follow-up. These results suggest a potential relationship between fluid balance and muscle ultrasound parameters. No causal inferences can be drawn; therefore, further studies are needed.

## Introduction

Intensive care unit-acquired weakness (ICU-AW) is a syndrome characterized by complications associated with the loss of muscle mass and function [1, 2]. Globally, between 25 and 65% of critically ill patients develop ICU-AW, particularly those with sepsis, multiorgan dysfunction, or prolonged mechanical ventilation [3, 4]. This condition affects up to 50% of patients ventilated for more than 7 days and is linked to higher mortality, extended ICU and hospital stays, and persistent reductions in quality of life [5]. In Chile, ICU-AW has been reported in 43% of survivors, contributing to prolonged hospitalization, difficult ventilator weaning, and long-term functional impairments [6].

Multiple risk factors contribute to ICU-AW, including immobility, deep sedation, systemic inflammation, organ failure, and hyperglycemia [7]. More recently, fluid overload has been proposed as a modifiable factor, as it may promote interstitial edema and disrupt muscle architecture [8]. Skeletal muscle wasting begins early in critical illness, with studies showing up to 2–3% loss of rectus femoris muscle size per day and up to 30% within the first 10 days of ICU stay [9].

Up to 86% of ICU patients experience a positive fluid balance during their stay, and approximately one-third are discharged while still fluid-overloaded [10]. Persistent fluid accumulation has been associated with increased mortality and worse functional outcomes [11]. In response, therapeutic strategies such as ultrafiltration (UF) have been implemented to help restore fluid balance. UF, typically delivered via continuous renal replacement therapy (CRRT), enables gradual fluid removal with limited hemodynamic compromise [12, 13]. AKI itself—particularly when requiring CRRT—has also been associated with accelerated muscle wasting, functional decline, and higher ICU-AW risk, likely through metabolic and inflammatory pathways as well as non-selective amino acid clearance [14]. Muscle ultrasound has emerged as a promising bedside tool to monitor changes in muscle architecture, offering a non-invasive and repeatable method to assess parameters, such as echogenicity and muscle thickness [9]. Prior studies suggest that fluid overload may lead to overestimated muscle thickness and altered echogenicity, potentially masking true muscle quality [15, 16]. Despite the growing use of UF in critically ill patients, limited data exist on its association with muscle ultrasound parameters or muscle function. This study aims to explore the relationship between fluid removal muscle performance in critically ill patients undergoing CRRT.

## Methods

### Study design

A prospective cohort study was conducted in the Intensive Care Unit (ICU) of Clínica Alemana de Santiago, with approval from the Institutional Ethics Committee (IRB #2012-53). The informed consent was waived in agreement with the observational nature of this study. The procedures were followed in accordance with the ethical standards of the responsible committee on human experimentation and with the Helsinki Declaration of 1975. Values of variables during the first 24 h of ICU admission were collected.

### Patient selection

We included critically ill adult patients (≥ 18 years) who underwent CRRT with UF. At the time UF was initiated, all patients met clinical criteria for resolved hypoperfusion, defined as adequate clinical perfusion and a lactate clearance ≥ 50%. The initiation and dosing of CRRT and UF were determined independently by the attending clinical team without involvement from the research team. Glycemic control in our unit is guided by a strict protocol that includes insulin therapy to maintain blood glucose levels within a target range of 140–180 mg/dL [17]. Patients were excluded if they had pre-existing neuromuscular disorders, leg amputations, functional limitations prior to admission, or if muscle ultrasound or strength assessments could not be performed due to clinical or logistical reasons.

### Muscle ultrasound assessment

Ultrasound images of the quadriceps (rectus femoris and vastus intermedius) were obtained with the patient in a supine position and the leg in passive extension. The transducer was placed perpendicular to the longitudinal axis of the thigh, at the midpoint between the anterior superior iliac spine and the superior border of the patella. This anatomical midpoint was consistently marked to improve reproducibility across time points. A high-frequency linear probe from a SonoSite Edge II (Fujifilm Sonosite Inc., Bothell, WA, USA) ultrasound system was used, maintaining fixed gain, depth, and machine settings across all measurements.

The following parameters were assessed:

- Rectus femoris thickness (RFT)
- Vastus intermedius thickness (VIT)
- Rectus femoris cross-sectional area (RF-CSA)
- Rectus femoris echogenicity (RFE)
- Subcutaneous tissue thickness (STT).

To quantify echogenicity, a 2 × 2 cm square region of interest (ROI) was selected in the mid-belly of the rectus femoris muscle, following the method described by Perry et al. Images were exported and analyzed using ImageJ software. The grayscale intensity histogram function was applied to compute the mean pixel intensity values within the ROI, on a scale ranging from 0 (black) to 255 (white) (1).

### Muscle strength assessment

Muscle strength was assessed using the Medical Research Council Sum Score (MRC-SS). Evaluations were performed in the morning (between 08:00 and 11:00 h) to reduce circadian variability. Six bilateral muscle groups were tested. All measurements (T2) were conducted in patients with a Richmond Agitation-Sedation Scale score of 0 and when they reached a score of ≥ 3 on the S5Q sedation scale, ensuring adequate alertness and cooperation for valid assessment.

### Physical therapy

All patients received daily physical therapy as part of the standard institutional early rehabilitation protocol, which included progressive passive or active mobilization depending on the patient’s clinical condition.

### Exploratory control cohort

In addition, we analyzed an exploratory control group of 10 critically ill patients undergoing CRRT without net ultrafiltration (UF = 0 mL/kg) during the same study period. These patients fulfilled the same inclusion and exclusion criteria as the UF cohort. Ultrasound and MRC-SS assessments followed identical procedures and time frames (*T*1 at CRRT initiation and *T*2 at 36 h). This analysis was performed post hoc and considered exploratory. Between-group differences in Δ (*T*2–*T*1) were evaluated using the Mann–Whitney U test. Hodges–Lehmann estimators with bootstrap 95% confidence intervals were calculated to quantify median differences. Given the small sample size, these analyses were descriptive and intended to contextualize the main results.

### Statistical analysis

Descriptive analysis was initially performed with results expressed as proportions for categorical variables or medians [25th–75th percentiles] for continuous variables. Normality of continuous variables was assessed using the Shapiro–Wilk test. The Wilcoxon signed-rank test was applied for paired samples. Correlations between ultrafiltration volume and functional improvement were evaluated using Spearman’s correlation coefficient (*ρ*). Receiver operating characteristic (ROC) curve analysis and Youden’s statistics were used to identify a UF volume threshold predictive of the probability of strength recovery. Statistical analysis was conducted using SPSS software, version 20 (SPSS Inc., Chicago, IL, USA), with statistical significance set at *p* < 0.05.

## Results

**Fig. 1:**
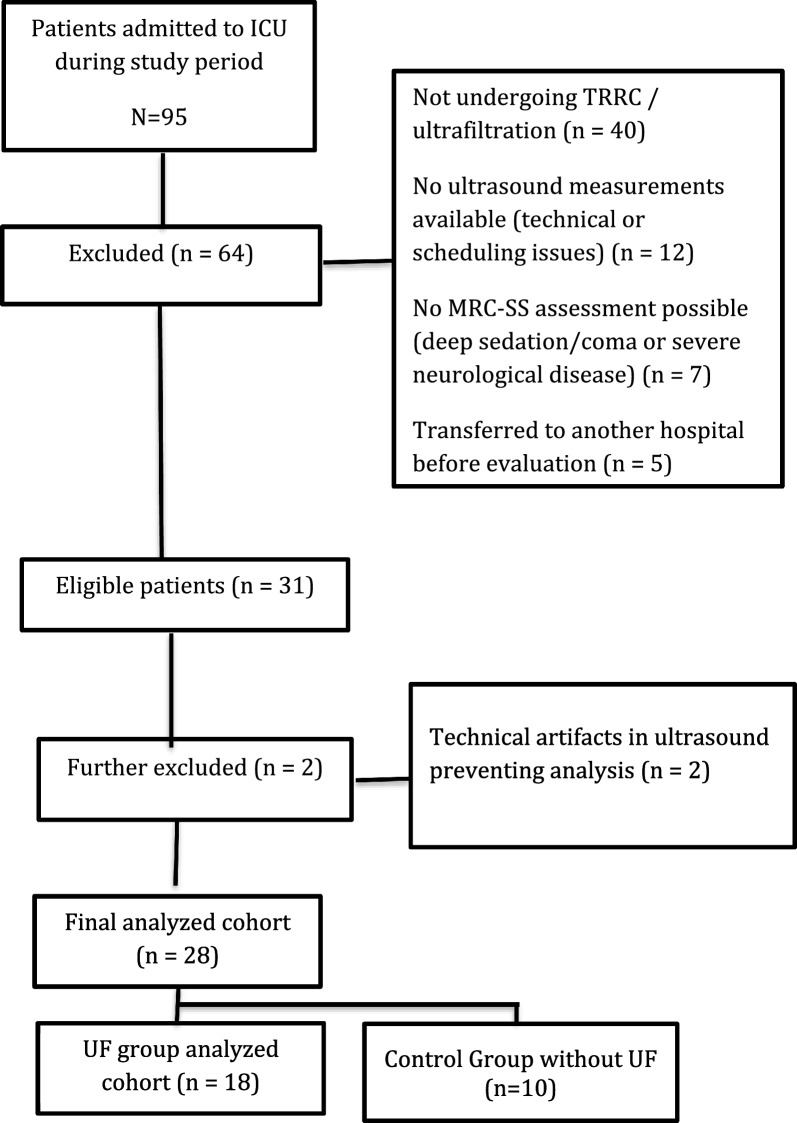
Flowchart

Twenty-eight critically ill patients undergoing CRRT were enrolled, eighteen of them critically ill with UF and 10 without UF (see Fig. 1).

They were mainly male with pneumonia and sepsis as ICU diagnoses. Baseline characteristics are shown in Table 1.

**Table 1:** Baseline characteristics of the study population

Variable	UF group (n = 18)	Control group (n = 10)
Age, years (median [IQR])	64 (55–72)	62 (54–70)
Sex	14 (77.8%) male/4 (22.2%) female	6(60%) male/4 (40%) female
APACHE II score	22 (18–26)	21 (17–25)
Days on MV before diagnosis	4 (3–6)	4 (3–5)
Pneumonia 7(38.9%)		4(40%)
Sepsis 5(27.8%)		3(25%)
Shock 4(22.2%)		2(20%)
COPD 1(5.6%)		1(15%)

Table 1 summarizes the baseline characteristics of critically ill patients included in the study. Continuous variable are reported as median and categorical variables are presented as counts and percentages

APACHE II score Acute Physiology and Chronic Health Evaluation II, SOFA Sequential Organ Failure Assessment and COPD chronic obstructive pulmonary disease

**Fig. 2:**
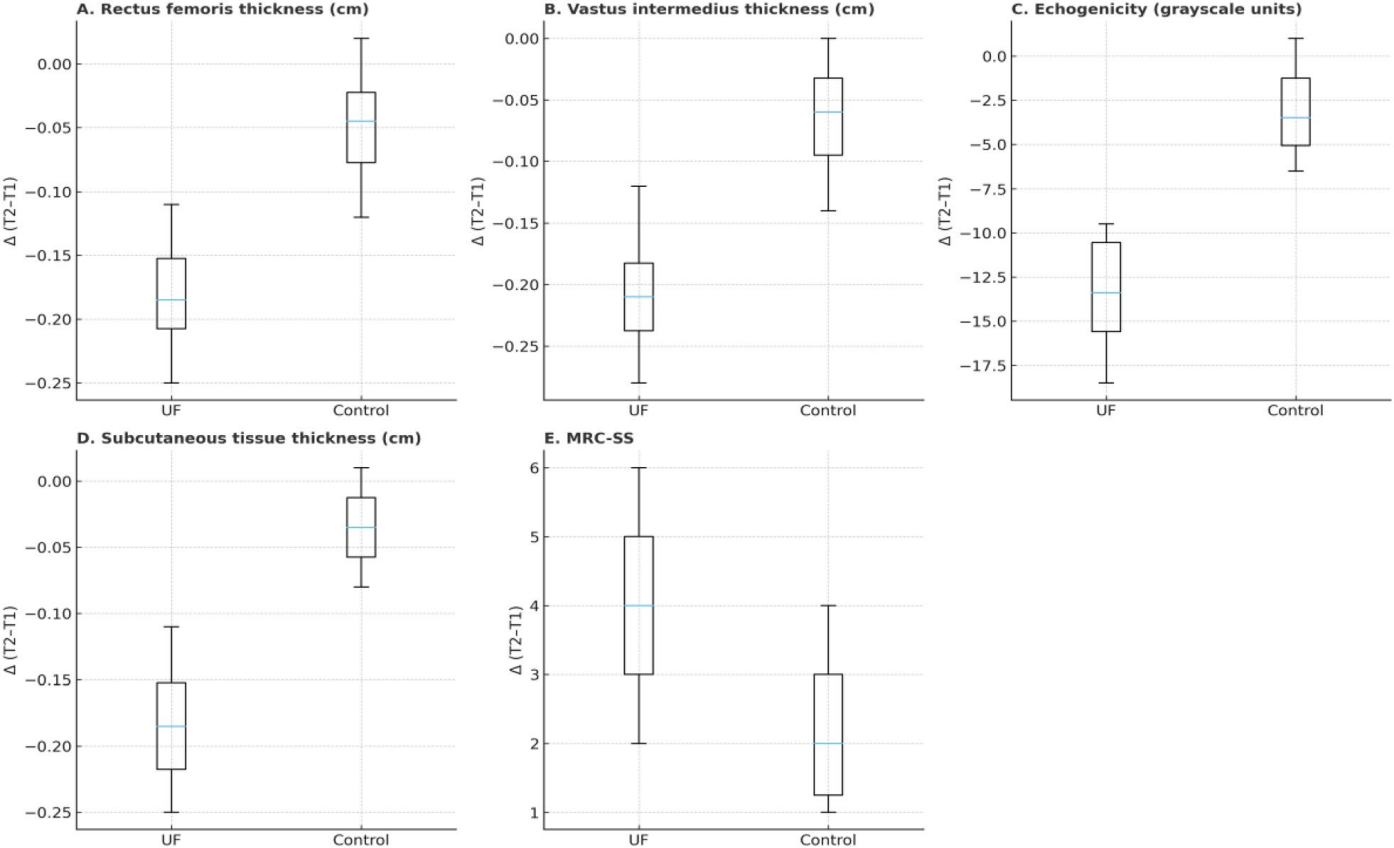
Changes in ultrasound and muscle strength parameters (UF vs. Control). **A** Rectus femoris thickness, **B** vastus intermedius thickness, **C** echogenicity, **D** subcutaneous tissue thickness, and **E** Medical Research Council Sum Score (MRC-SS). Boxplots display medians with interquartile ranges. UF = ultrafiltration; MRC-SS = Medical Research Council Sum Score

The control group showed minimal changes in ultrasound parameters and strength (Fig. 2).

The ultrasound and MRC findings between UF and control groups are shown in Table 2. We found a significant difference in all ultrasound variables while MRC had a lack of difference.

**Table 2:** Between-group differences in ultrasound and strength changes (Δ*T*2–*T*1)

Parameter	UF group Δ (median, IQR)	Control Δ (median, IQR)	Δ difference UF–Control (Hodges–Lehmann [95% CI])	*p*-value
Rectus femoris thickness (cm)	– 0.17 (– 0.25 to – 0.09)	– 0.05 (– 0.12 to + 0.02)	– 0.11 [– 0.20 to – 0.04]	0.01
Vastus intermedius thickness (cm)	– 0.19 (– 0.28 to – 0.12)	– 0.07 (– 0.14 to 0.00)	– 0.10 [– 0.19 to – 0.03]	0.01
Echogenicity (grayscale units)	– 14.0 (– 18.5 to – 9.5)	– 3.0 (– 6.5 to + 1.0)	– 10.5 [– 15.8 to – 6.2]	< 0.001
Subcutaneous tissue thickness (cm)	– 0.18 (– 0.25 to – 0.11)	– 0.04 (– 0.08 to + 0.01)	– 0.13 [– 0.19 to – 0.07]	< 0.001
MRC-SS	+ 4.0 (+ 2.0 to + 6.0)	+ 2.0 (+ 1.0 to + 4.0)	+ 1.5 [– 1.0 to + 4.0] (NS)	0.18

Abbreviations: UF = ultrafiltration; IQR = interquartile range; CI = confidence interval; MRC-SS = Medical Research Council Sum Score

**Fig. 3:**
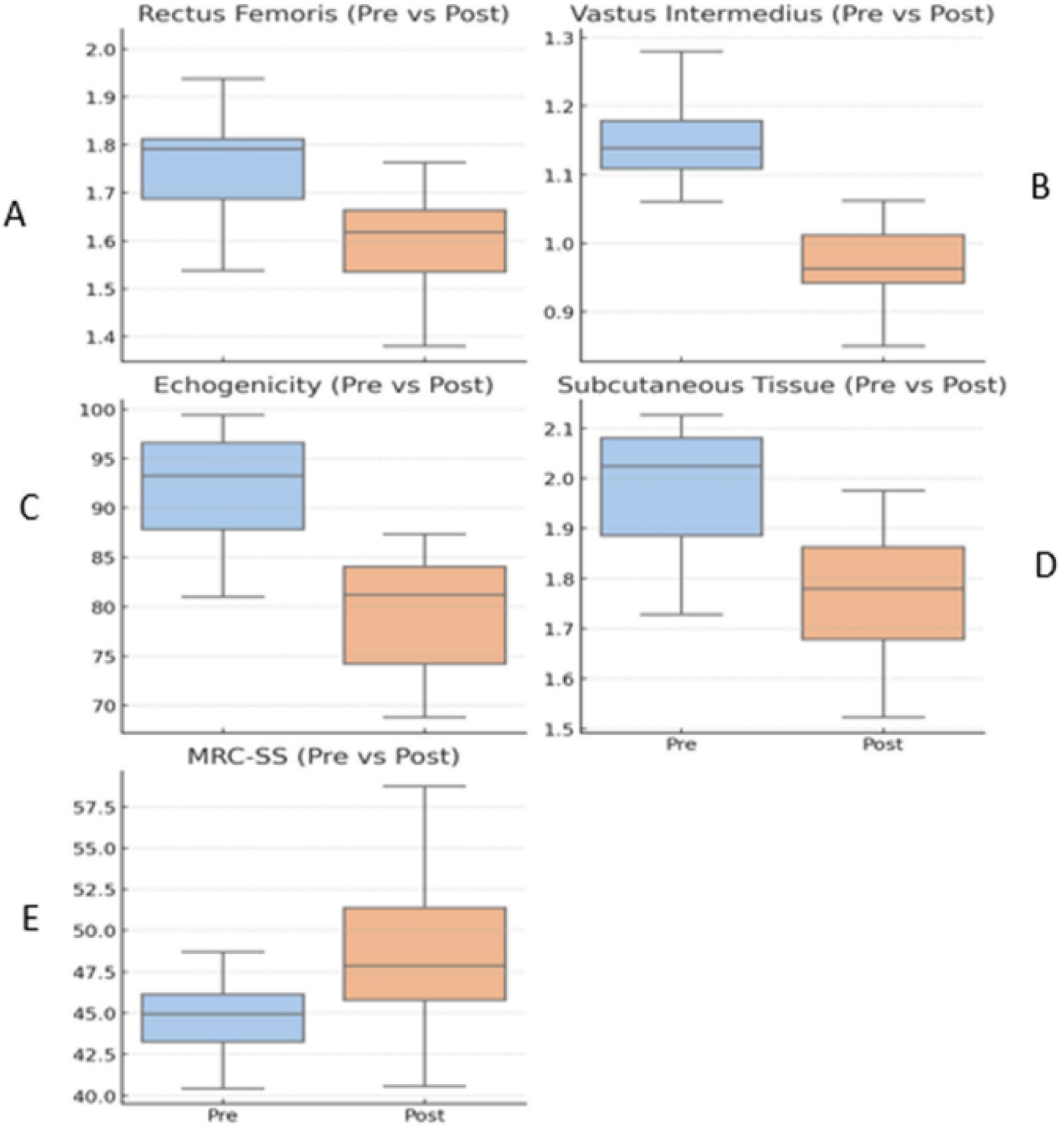
Patients' UF group. Changes in rectus femoris thickness, vastus intermedius thickness, rectus femoris echogenicity, subcutaneous tissue thickness, and MRC-SS between baseline (T1) and follow-up (T2). Values shown as medians with interquartile ranges. Abbreviations RFT = rectus femoris thickness, VIT = vastus intermedius thickness, RFE = rectus femoris echogenicity, STT = subcutaneous tissue thickness, MRC-SS = Medical Research Council Sum Score

In the UF group, ultrasound measurements revealed significant reductions in muscle and subcutaneous parameters between the two time points. Median rectus femoris thickness decreased from 1.74 to 1.57 cm (*p* = 0.03), and vastus intermedius from 1.14 to 0.95 cm (*p* < 0.01). Muscle echogenicity declined from 91.7 to 78.3 grayscale units (*p* < 0.01), while subcutaneous tissue thickness was reduced from 1.98 to 1.79 cm (*p* < 0.01). Global muscle strength, assessed by the MRC-SS, increased from 45.0 to 49.0 points (*p* = 0.05). These changes are shown in Fig. 3.

**Fig. 4:**
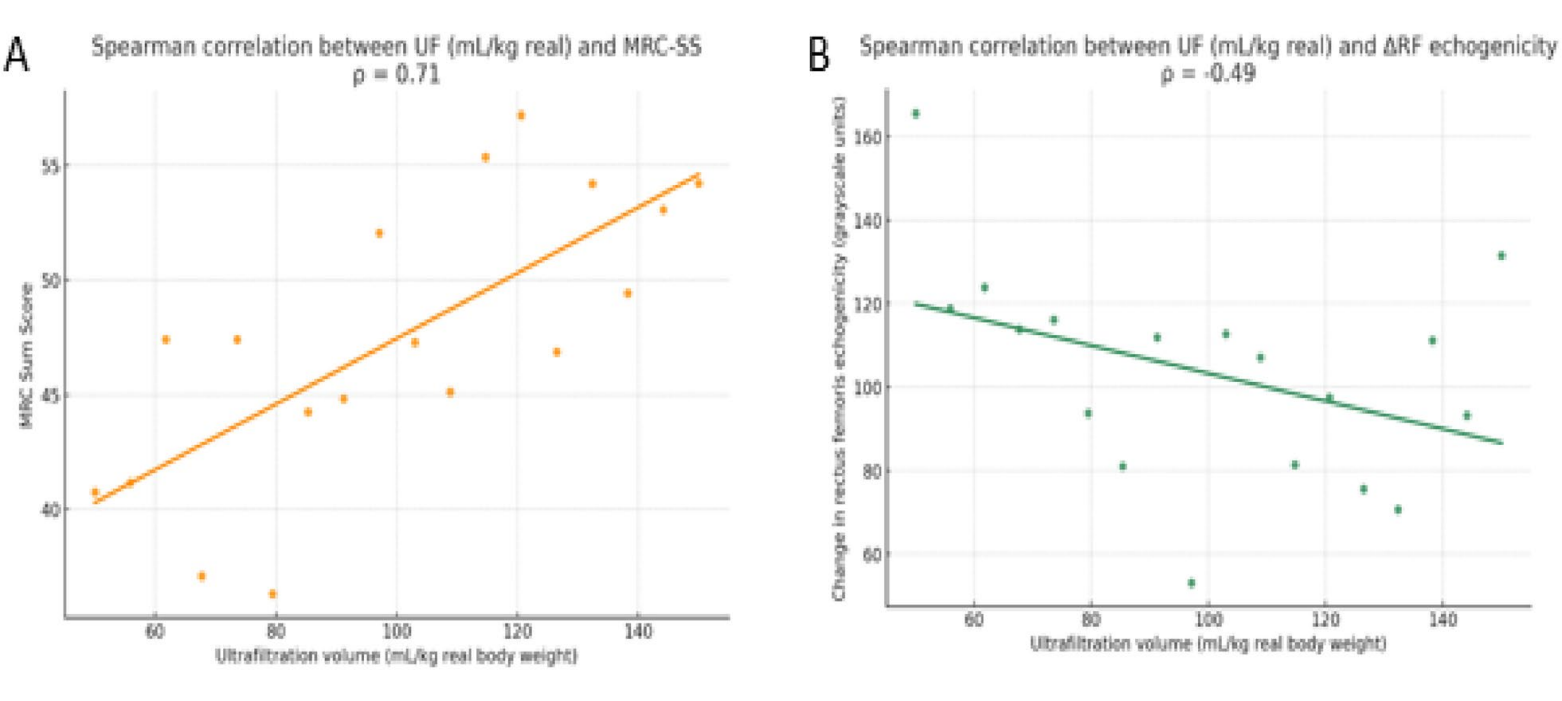
Correlation between ultrafiltration (UF) volume (mL/kg) and **A** muscle strength at T2 (MRC-SS) and **B** change in rectus femoris echogenicity. Spearman’s correlation coefficients are shown. Abbreviations UF = ultrafiltration; MRC-SS = Medical Research Council Sum Score

In patients of the UF group, correlation analyses revealed a statistically significant positive association between ultrafiltration volume (mL/kg) and muscle strength at T2 (Spearman’s *ρ* = 0.71, *p* < 0.001), and a negative association between UF volume and change in rectus femoris echogenicity (Spearman’s *ρ* = – 0.49, *p* = 0.039) (Fig. 4). No significant correlations were found between UF volume and changes in muscle thickness (rectus femoris or vastus intermedius), cross-sectional area, or subcutaneous tissue thickness.

**Fig. 5:**
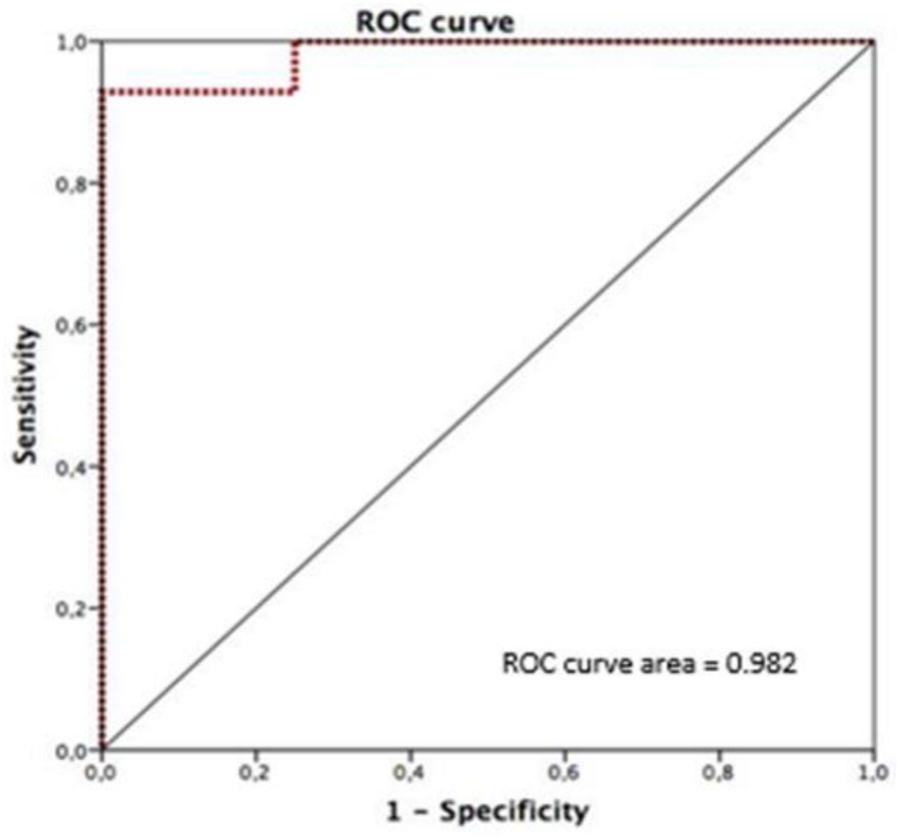
Receiver operating characteristic (ROC) curve analysis for UF volume (mL/kg) and probability of strength recovery (MRC-SS > 48). The area under the curve (AUC) with a 95% confidence interval is shown. Abbreviations: UF = ultrafiltration; ROC = receiver operating characteristic; AUC = area under the curve; MRC-SS = Medical Research Council Sum Score

The area under the ROC curve (AUC) for UF volume and probability of strength recovery was 0.982 (95% CI: 0.928–1.000; *p* = 0.004), with an optimal cutoff of 82.12 mL/kg yielding 92.9% sensitivity and 100% specificity (Fig. 5). This threshold was considered exploratory only and does not replace the established definition of ICU-acquired weakness (MRC-SS ≤ 48/60).

**Fig. 6:**
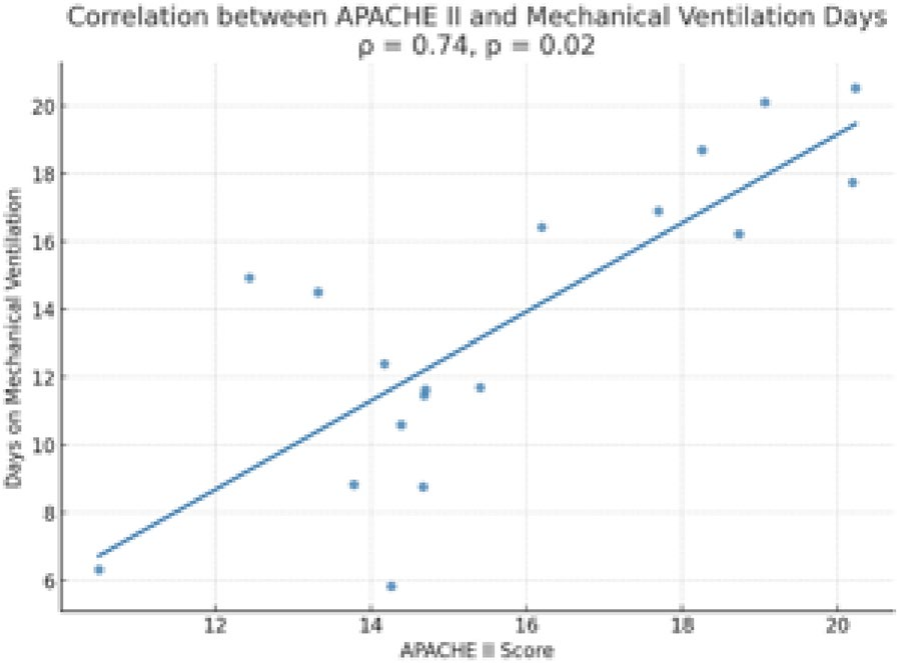
Correlation between APACHE II score and number of days on mechanical ventilation. Spearman’s correlation coefficient is shown. Abbreviations: APACHE II = Acute Physiology and Chronic Health Evaluation II

Additionally, a statistically significant positive correlation was found between APACHE II scores and the number of days on mechanical ventilation (Spearman’s *ρ* = 0.74, *p* = 0.02; see Fig. 6).

## Discussion

In this pilot study, fluid removal through ultrafiltration (UF) was consistently associated with acute changes in muscle ultrasound parameters, specifically muscle echogenicity and subcutaneous tissue thickness. These findings may be compatible with a reduction in interstitial and subcutaneous edema, which are common in critically ill patients experiencing fluid overload. Echogenicity is a non-invasive marker of tissue quality, and its reduction following UF may reflect decreased fluid content, resulting in improved structural definition in ultrasound imaging. Similarly, the observed decrease in subcutaneous tissue thickness may indicate extracellular fluid clearance. These consistent changes across ultrasound metrics highlight the potential role of point-of-care ultrasound in dynamically monitoring fluid-related tissue alterations in the intensive care setting.

Our results align with previous studies by Cheng et al. and Stanley et al., which reported associations between fluid status and ultrasound-based muscle characteristics. Together, these observations suggest that fluid shifts may influence ultrasonographic muscle parameters, regardless of functional outcomes [15, 16]. In contrast, Mayer et al. (2025) reported a significant increase in muscle echogenicity and a progressive reduction in rectus femoris thickness and cross-sectional area in patients with acute kidney injury undergoing continuous kidney replacement therapy during the first week of hospitalization [14]. In their study, these changes were interpreted as evidence of structural deterioration due to muscle catabolism. Conversely, our cohort showed a decrease in muscle echogenicity and subcutaneous tissue thickness, which may reflect improved ultrasound delineation of muscle architecture associated with extracellular fluid removal through UF. This divergence may be explained by differences in the clinical timing of the assessments: while Mayer and colleagues evaluated patients in a more prolonged and catabolic phase of critical illness, our assessments were performed after resolution of hypoperfusion, when interstitial edema may be more prominent. Importantly, the exploratory control group analyzed in our study showed minimal changes in ultrasound and strength parameters, supporting the interpretation that the acute reductions observed in the UF cohort were related to fluid removal rather than spontaneous recovery. Taken together, these observations suggest that muscle ultrasound parameters may be influenced by both structural and fluid-related factors, and that UF could contribute to improved sonographic definition of muscle tissue in fluid-overloaded patients, without necessarily implying structural or functional improvement of the muscle itself. In this context, muscle ultrasound may serve as a useful bedside tool to dynamically monitor tissue changes in response to fluid management interventions in the ICU.

Although an increase in global muscle strength (MRC-SS) was observed after UF, this finding should be considered exploratory. No significant correlation was found between UF volume and the change in MRC-SS (ΔMRC-SS), and the known interobserver variability of this scale, as reported by Connolly et al., may explain the modest difference observed. In addition, although MRC-SS assessments were performed only when patients reached S5Q ≥ 3, residual sedation may still have influenced strength scores [18]. Furthermore, the minimal clinically important difference (MCID) for MRC-SS has been estimated at 4 points (Fossat et al., JAMA 2018) [19], and the changes observed in our study were below this threshold. Therefore, improvement in strength cannot be attributed to fluid removal alone.

The primary focus of this study lies in the acute ultrasound-detected changes, which were objective, quantifiable, and clinically plausible responses to a fluid management intervention. This approach is particularly relevant for sedated or non-communicative patients, where traditional strength assessments may not be feasible. Strengths of this study include the use of validated assessment tools (muscle ultrasound and MRC-SS), standardized measurement methods and timing.

## Limitations

This study has several limitations. First, the small sample size and pilot nature precludes the generalizability of our findings. Second, although an exploratory control group was included in the supplementary analysis, the main study design was self-controlled (T1–T2 comparisons), which reduces inter-patient variability but precludes causal inference. Third, several unmeasured confounders—such as central venous pressure or cardiac dysfunction—may have influenced both ultrasound and functional outcomes. Fourth, despite marking the anatomical midpoint between the anterior superior iliac spine and the superior patellar border, ultrasound remains an operator-dependent technique, and small differences in probe inclination or pressure may have introduced variability. Finally, residual sedation may have affected strength scores despite standardized evaluation conditions, and the changes observed in MRC-SS did not exceed the minimal clinically important difference (MCID) of 4 points [19].

## Future directions

Future studies should aim to confirm these preliminary associations in larger, multicenter cohorts, ideally including parallel control groups to strengthen causal inference. Incorporation of objective tools to quantify fluid status (e.g., bioimpedance or dilution techniques) may help disentangle the contribution of fluid shifts from structural muscle changes. Longitudinal monitoring of muscle echogenicity and strength throughout the ICU stay could clarify the temporal relationship between fluid balance, muscle quality, and outcomes. Finally, evaluating the prognostic value of muscle ultrasound parameters in predicting clinically relevant outcomes—including weaning success, ICU length of stay, and post-ICU functional status—represents a promising area for future research.

## Conclusion

In this observational pilot study, ultrafiltration was consistently associated with acute reductions in muscle echogenicity and subcutaneous tissue thickness, as well as a modest improvement in global muscle strength among critically ill patients. These findings suggest a potential link between fluid removal and improved ultrasound delineation of muscle architecture, although causality cannot be inferred. The consistent ultrasound-based changes following UF highlight the value of point-of-care muscle ultrasound as a dynamic, non-invasive tool to monitor tissue-level responses to fluid management in the ICU. Importantly, the exploratory control group showed minimal changes, supporting the interpretation that UF, rather than spontaneous recovery, underlies the observed ultrasound differences. This pilot study contributes to the growing research in the interplay between fluid balance and ICU-acquired weakness. Future research should confirm these findings in larger controlled trials, evaluate their prognostic relevance for functional recovery, and determine whether tailored fluid removal strategies can be integrated into early rehabilitation protocols for critically ill patients.

## Data Availability

The datasets generated and/or analyzed during the current study are not publicly available due to ethical restrictions concerning patient privacy and confidentiality but are available from the corresponding author on reasonable request.
